# Effects of Exercise Training on Neurotrophic Factors and Blood–Brain Barrier Permeability in Young-Old and Old-Old Women

**DOI:** 10.3390/ijerph192416896

**Published:** 2022-12-16

**Authors:** Su-Youn Cho, Hee-Tae Roh

**Affiliations:** 1 Exercise Physiology Laboratory, Department of Physical Education, Yonsei University, Seoul 03722, Republic of Korea; 2 Department of Sports Science, College of Health Science, Sun Moon University, 70 Sunmoon-ro 221 beon-gil, Tangjeong-myeon, Asan-si 31460, Republic of Korea

**Keywords:** elderly, exercise, oxidative stress, sirtuin, neurotrophin, blood–brain barrier

## Abstract

Aging and regular exercise may have opposite effects on brain health, and although oxidative stress and sirtuins may be involved in these effects, studies on this topic are limited. Accordingly, the present study aimed to verify the effect of exercise training on oxidant–antioxidant balance, neurotrophic factors, blood–brain barrier permeability, and sirtuins in young-old and old-old women. The study participants were 12 women aged 65–74 years (Young-Old group) and 12 women aged 75–84 years (Old-Old group). All of the selected participants performed exercise training consisting of treadmill walking and resistance band exercise three times a week for 12 weeks. Blood samples were collected before and after exercise training to analyze serum oxidant–antioxidant markers (reactive oxygen species [ROS], superoxide dismutase [SOD]), neurotrophic factor (brain-derived neurotrophic factor [BDNF], vascular endothelial growth factor [VEGF]) levels, and blood–brain barrier permeability marker (S100 calcium-binding protein β [S100β], matrix metalloproteinase-9 [MMP-9]) levels, and sirtuin (SIRT-1, SIRT-2, SIRT-3) levels. The Young-Old group showed significantly increased SOD, BDNF, VEGF, SIRT-1, and SIRT-3 levels after training in comparison with the levels before training (*p* < 0.05), and a significantly higher BDNF level than the Old-Old group after training (*p* < 0.05). On the other hand, the Old-Old group showed significantly higher SIRT-1 levels after training in comparison with the levels before training (*p* < 0.05). Thus, exercise training may be effective in increasing the levels of neurotropic factors and reducing blood–brain barrier permeability in the elderly women, and increased antioxidant capacity and elevated levels of sirtuins are believed to play a major role in these effects. The positive effect of exercise may be greater in participants of relatively young age.

## 1. Introduction

Old age is characterized by a decline in physical, psychological, and cognitive capacities and an increased possibility of chronic diseases [1,2]. Cognitive decline is one of the biggest health problems in the elderly. Cognitive functions, including memory, learning, attention, perception, reasoning, and problem-solving abilities, gradually deteriorate with age and eventually lead to difficulties in normal daily life [3,4]. The aging of the brain is caused by the increased generation of reactive oxygen species (ROS) as a result of deficiencies in the in vivo antioxidant defense system and increased oxidative stress (OS) [5]. The brain is easily exposed to OS-induced apoptosis because of its high oxygen utilization rate in comparison with other tissues and the presence of many unsaturated fatty acids that are easy to oxidize, while the antioxidant defense system is weak and complex chemical reactions occur continuously in the brain to facilitate the production of various neurotransmitters. In addition, excessive OS can suppress the expression of neurotrophins such as brain-derived neurotrophic factor (BDNF), which is an important neurotrophic factor that regulates brain learning, memory, and synaptic functions, and causes negative changes in brain perfusion and blood–brain barrier (BBB). Such changes are also suggested to be responsible for lowering cognitive function in the elderly and causing cerebrovascular aging and related diseases [6,7,8].

On the other hand, regular exercise strengthens the brain blood circulation and antioxidant enzyme system, and promotes the secretion of neurotrophins, which can have a positive effect on maintaining and improving cognitive function in the elderly [9,10,11]. Some researchers have reported that exercise can increase neuronal survival and cognitive ability by increasing mitochondrial biosynthesis and enzymes related to oxidative phosphorylation, which are associated with increased sirtuin (SIRT) activity [12,13].

SIRTs are proteins with nicotinamide adenine dinucleotide-dependent deacetylase or adenosine diphosphate ribosyl transferase activity, and seven SIRTs, from SIRT-1 to SIRT-7, have been identified. They play important roles in the pathogenesis of age-related diseases such as cancer, diabetes, cardiovascular diseases, and neurological diseases [14,15]. SIRT activity has been reported to be related to various cellular metabolisms related to aging, such as mitochondrial ROS generation, BBB permeability, antioxidant function, and neurogenesis [15]. Several previous studies have shown that regular exercise induces cellular metabolic stress that affects SIRTs and induces positive changes in the activity or expression of SIRTs, improving the efficiency of oxidative metabolism, thereby enhancing biogenesis and mitochondrial function and maintaining the antioxidant system [16,17,18]. However, these studies had limitations in that most of them were conducted on animal and cell models, and some studies conducted on human participants mainly involved healthy individuals, with very few studies reporting such effects in the elderly. 

Health-related problems appear more frequently in old-old individuals over 75 years of age in comparison with young-old individuals under 74 years of age [19,20]. In addition, the positive effects of regular exercise have been suggested to differ depending on the degree of aging [21]. Therefore, to compose an exercise program for the health of the elderly in an aging society, elderly individuals over 65 years of age should be classified into groups based on distinct age ranges and not pooled into a single group, and exercise methods suitable for the characteristics of each group should be proposed. Accordingly, the purpose of this study was to examine the effects of regular exercise training on oxidant–antioxidant balance, neurotrophic factors, BBB permeability, and SIRTs by dividing the elderly into Young-Old and Old-Old groups.

## 2. Methods

### 2.1. Study Design and Participants

This study was a prospective, randomized, controlled, parallel, and two-group trial. This prospective intervention was conducted with 24 elderly women (age, 73.83 ± 5.21 years; height, 153.40 ± 4.86 cm; weight, 57.71 ± 5.70 kg; and body mass index [BMI], 24.50 ± 1.93 kg/m^2^) aged 65–84 years residing in the local community who voluntarily wished to participate in this study. The eligibility criteria for participation in this study were age 65–74 years (Young-Old group, *n* = 12) or 75–84 years (Old-Old group, *n* = 12) without physical dysfunction or musculoskeletal disease that could limit exercise participation. We excluded smokers, obese people with a BMI of 30 or higher, individuals participating in a regular exercise program, and patients with cardiopulmonary diseases or neurological or cognitive disorders. All participants were fully explained about the benefits and risks of participating in the study, and their written informed consent was obtained. The protocol of this study was approved by the National Research Foundation of Korea (NRF-2017R1C1B5017956), and the study conformed to the standards set by the latest revision of the Declaration of Helsinki. Baseline biochemical and anthropometric characteristics of the participants are shown in Table 1.

### 2.2. Anthropometric Measurements

Anthropometric measurements, including height, weight, and BMI, were obtained. Height was measured to the nearest 0.1 cm on a semiautomatic height measurement equipment (HD; STDK, Tokyo, Japan); weight was measured to the nearest 0.1 kg on a medical balance (GL-6000-20; CAS, Seoul, Republic of Korea). BMI was calculated by dividing the weight (kg) by the height squared (m^2^).

### 2.3. Blood Collection and Analysis Methods

Blood was collected from the antecubital vein before and after exercise training by using a 22-gauge needle and transferred to serum separation tubes; blood collection was performed in a resting state in which fasting had been maintained for more than 12 h. The collected blood was centrifuged at 3000 rpm for 15 min, and serum was separated and stored frozen (−80 °C) until analysis of oxidant–antioxidant markers (ROS, superoxide dismutase [SOD]), neurotrophic factors (BDNF, vascular endothelial growth factor [VEGF]), BBB permeability markers (S100 calcium-binding protein β [S100β], matrix metalloproteinase-9 [MMP-9]), and sirtuins (SIRT-1, SIRT-2, SIRT-3). Serum ROS was analyzed using the OxiSelect™ In Vitro ROS/RNS Assay Kit (#STA-347; Cell Biolabs, San Diego, CA, USA). In this assay 2′,7′-dichlorodihydrofluorescein is converted to 2′,7-dichlorodihydrofluorescein diacetate by ROS. Fluorescence was measured at 480 nm excitation/530 nm emission using a microplate reader (spectramax M2e; Molecular Devices, San Jose, CA, USA). Serum SOD activity was analyzed using a colorimetric assay with the Superoxide Dismutase Assay Kit (#706002; Cayman Chemicals, Ann Arbor, MI, USA) at 450 nm with a microplate reader. The serum BDNF, VEGF, S100β, MMP-9, SIRT-1, SIRT-2, and SIRT-3 levels were measured by the following commercially available enzyme-linked immunosorbent assay (ELISA) kits: Human/Mouse BDNF DuoSet ELISA kit (#DY248; R&D Systems, Minneapolis, MN, USA), Human VEGF DuoSet ELISA kit (#DY293B-05; R&D Systems, Minneapolis, MN, USA), Human S100B DuoSet ELISA kit (#DY1820-05; R&D Systems, Minneapolis, MN, USA), Human MMP-9 DuoSet ELISA kit (#DY911; R&D Systems, Minneapolis, MN, USA), Human Sirtuin 1 ELISA Kit (#CSB-E15058h; CUSABIO, Wuhan, China), Human Sirtuin 2 ELISA Kit (#MBS2530369; Mybiosource, San Diego, CA, USA), and Human Sirtuin 3 ELISA Kit(#CSB-EL021341HU; CUSABIO, Wuhan, China), respectively. During the calculations, the absorbance values of the standards and samples were read at 450 nm by using a spectrophotometer (Tecan Sunrise, TECAN GmbH, Salzburg, Austria).

### 2.4. Exercise Training Method

Exercise training consisted of 10 min each of warm-up and cool-down stretching and 60 min of main exercise, which included an endurance exercise session (30 min) and a resistance exercise session (30 min), for a total of 80 min of exercise. The program was conducted three times per week for 12 weeks. For the endurance exercise session, treadmill walking was performed for 30 min at rating of perceived exertion (RPE) 10–14 exercise intensity. For the resistance exercise session, 13 types of resistance band exercises (Alternate arm and leg lift, Charles Chaplin, Clam, Curl up, Hundred, Rockets, Rolling like a ball, Leg up and down, Shoulder bridge, Side knee open, Side double leg lift, Swan, Swimming) using an elastic band (spoband 25, spoband Co., Republic of Korea) were performed at RPE 10–14 exercise intensity.

### 2.5. Statistical Analyses

For data analysis, the mean and standard deviation of all dependent variables were calculated using SPSS version 26.0 for Windows (SPSS Inc., Chicago, IL, USA). The time and group differences in each dependent variable were verified using two-way repeated-measures analysis of variance (ANOVA). For statistically significant interaction effects, independent-sample *t*-test and paired-sample *t*-test were performed. All statistical significance levels (α) were set at 0.05.

## 3. Results

### 3.1. Changes in Serum Oxidant–Antioxidant Markers

**Figure 1:**
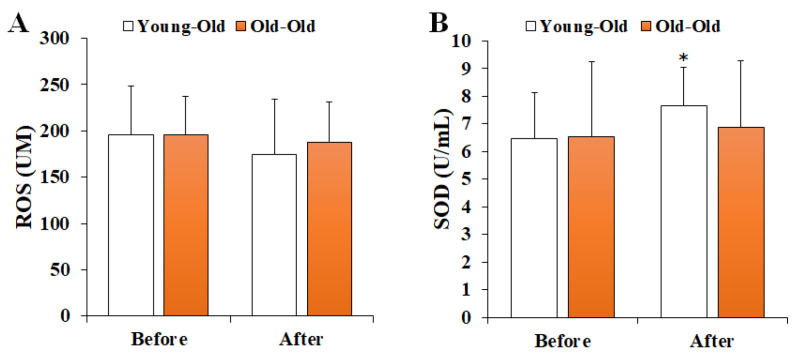
Changes in serum oxidant–antioxidant markers. Values presented by mean and standard deviation. (**A**) ROS, reactive oxygen species; (**B**) SOD, superoxide dismutase; * Significantly different versus Before (*p* < 0.05).

Figure 1 shows the changes in serum oxidant–antioxidant markers before and after 12 weeks of exercise training. Two-way repeated-measures ANOVA for the oxidant–antioxidant balance showed a significant interaction effect between time and group in SOD levels (*F* = 4.356, *p* = 0.049). The post hoc analysis showed no significant difference in the Old-Old group (*p* > 0.05), whereas the SOD level after training in the Young-Old group was significantly greater than the levels before training (*p* < 0.05). On the other hand, the ROS level (*F* = 0.999, *p* = 0.328) did not show a significant interaction effect.

### 3.2. Changes in Serum Neurotrophic Factor Levels

**Figure 2:**
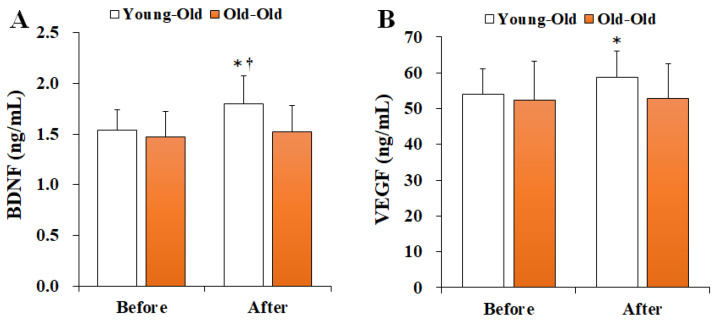
Changes in serum neurotrophic factor levels. Values presented by mean and standard deviation. (**A**) BDNF, brain-derived neurotrophic factor; (**B**) VEGF, vascular endothelial growth factor; * Significantly different versus Before (*p* < 0.05); ^†^ Significantly different versus Old-Old group (*p* < 0.05).

Figure 2 shows the changes in serum neurotrophic factor levels before and after 12 weeks of exercise training. Two-way repeated-measures ANOVA of neurotrophic factors showed a significant interaction effect between time and group in BDNF (*F* = 5.382, *p* = 0.030) and VEGF (*F* = 4.872, *p* = 0.038) levels. Post hoc analysis showed no significant difference in the Old-Old group (*p* > 0.05), whereas the BDNF and VEGF levels in the Young-Old group post training were significantly greater than those before training (*p* < 0.05). Moreover, the post training BDNF level in the Young-Old group was significantly higher than that in the Old-Old group (*p* < 0.05).

### 3.3. Changes in Serum BBB Permeability Markers

**Figure 3:**
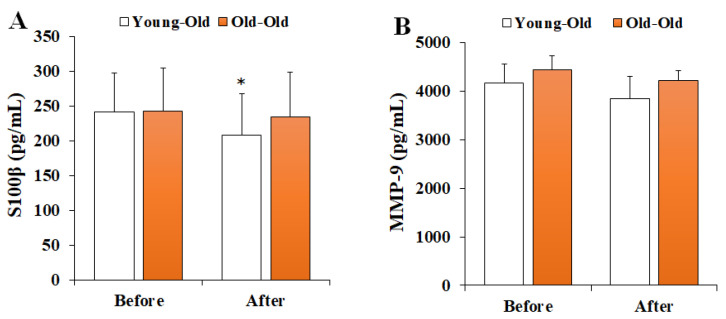
Changes in serum BBB permeability markers. Values presented by mean and standard deviation. (**A**) S100β, S100 calcium-binding protein β; (**B**) MMP-9, matrix metalloproteinases-9; * Significantly different versus Before (*p* < 0.05).

Figure 3 shows the changes in serum BBB permeability markers before and after 12 weeks of exercise training. Two-way repeated-measures ANOVA of BBB permeability showed a significant interaction effect between period and group in S100β levels (*F* = 5.194, *p* = 0.033). Post hoc analysis showed significantly decreased S100β levels after training in comparison with the levels before training in the Young-Old group (*p* < 0.05), whereas no significant difference was observed in the Old-Old group (*p* > 0.05). However, MMP-9 levels did not show a significant interaction effect (*F* = 1.408, *p* = 0.248).

### 3.4. Changes in Serum SIRTs Levels

**Figure 4:**
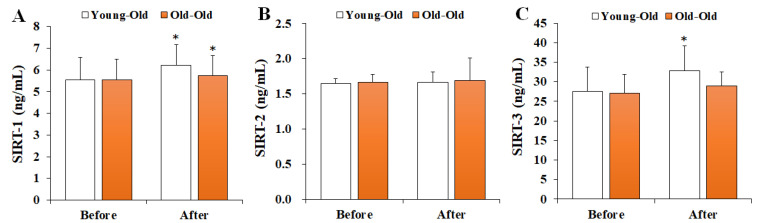
Changes in serum SIRTs levels. Values presented by mean and standard deviation. (**A**) SIRT-1, sirtuin-1; (**B**) SIRT-2, sirtuin-2; (**C**) SIRT-3, sirtuin-3; * Significantly different versus Before (*p* < 0.05).

Figure 4 shows the changes in serum SIRTs levels before and after 12 weeks of exercise training. Two-way repeated-measures ANOVA of SIRTs levels showed significant interaction effects between time and group for the SIRT-1 (*F* = 6.635, *p* = 0.017) and SIRT-3 (*F* = 6.542, *p* = 0.018) levels. In the post hoc analysis, SIRT-1 levels significantly increased in the Young-Old and Old-Old groups after training in comparison with the levels before training (*p* < 0.05), whereas SIRT-3 levels increased significantly only in the Young-Old group (*p* < 0.05). On the other hand, SIRT-2 levels did not show a significant interaction effect (*F* = 0.003, *p* = 0.958).

## 4. Discussion

The ROS levels continuously generated during aerobic metabolism vary depending on the cell type, cell age, ROS exposure history, and other factors and may be escalated during aging. In addition, with increasing age, energy production in the brain decreases and the levels of ROS such as O_2_^−^ and H_2_O_2_ in mitochondria increase, while the activity of antioxidant defense enzymes such as SOD decreases [22,23,24]. Thus, aging converts the redox status from antioxidant to pro-oxidant status, which may negatively influence the brain aging process and the pathogenesis of various neurodegenerative diseases. However, regular exercise can increase resistance to OS through regulation of the redox status and aging-related structural and functional changes in the brain through increased levels of neurotrophic factors and neurogenesis, increased capillary blood vessels, decreased oxidative damage, and increased proteolysis. Exercise has been shown to have a beneficial effect on the brain and to play an important role in the prevention and treatment of cerebrovascular diseases [9,10,11,12,13,25].

In this study, the effects of 12 weeks of exercise training on serum oxidant–antioxidant markers in young-old and old-old individuals were investigated. The SOD levels were significantly increased in the Young-Old group. SOD, the representative protective system involved in antioxidant cellular defense, catalyzes the transformation of peroxides into oxygen and hydrogen peroxide. However, during the course of aging, the expression and activity of the SOD system may be altered, reducing the cellular ability to fight oxidant molecules and, consequently weakening resistance to ROS accumulation [26]. However, exercise has been reported to improve the oxidation/antioxidant balance by activating pathways involved in the transcription of antioxidant gene enzymes and enhancing the antioxidant defense system by increasing resistance to cellular stress [21,25,27]. Kozakiewicz et al., reported that the activity of antioxidant enzymes, including SOD, was higher in the physically active elderly than in the inactive elderly [25]. In addition, Bouzid et al., showed that the activity levels of SOD and antioxidant enzymes were lower in the elderly than in young people [21]. However, their study showed no difference in SOD activity between the elderly who exercised regularly and the younger people who were inactive, suggesting that regular exercise could prevent age-related decline in antioxidant defenses.

SIRTs are crucial mediators in the exercise-related changes in the activity of antioxidant enzymes. SIRTs play important roles in many cellular functions, including histone deacetylation and protein acylation and deacetylation [28]. The SIRT family consisting of seven enzymes is reported to be involved in several antioxidant and OS-related processes and functions, including longevity, mitochondrial function, DNA damage repair, and overall metabolism [15]. SIRT-1, SIRT-3, and SIRT-5 protect cells from ROS; SIRT-2, SIRT-6, and SIRT-7 regulate key OS genes and mechanisms; and SIRT-4 induces ROS production and also acts as an antioxidant [29]. Exercise can increase the expression and activity of SIRTs by inducing cellular metabolic stress that affects SIRTs, and studies mainly related to SIRT-1 and SIRT-3 have been reported in this regard. Bayod et al., reported that the protein content of SIRT-1 and peroxisome proliferator-activated receptor γ co-activator 1α increased in rat muscle after 36 weeks of treadmill training [16], and Hokari et al., reported that while SIRT-3 content was increased in rat skeletal muscle after four weeks of voluntary exercise or treadmill training, it was downregulated in fixed soleus muscle [30]. In addition, Koltai et al., reported that skeletal muscle SIRT-1, SIRT-3, and SOD2 levels of master athletes aged 65 years and older were higher than those of the control group of the same age [31], and Johnson et al., reported that eight weeks of endurance training increased the expression of skeletal muscle SOD2 and SIRT-3 in both the young and the elderly [32]. In our study, after a 12-week exercise program, SIRT-1 and SIRT-3 levels in the Young-Old group and SIRT-1 levels in the Old-Old group increased significantly. These results support the findings of previous studies showing that regular exercise can inhibit the deterioration of the antioxidant system caused by aging.

An increase in antioxidant system function following regular exercise may also influence aging-related changes in the BBB. The BBB is a multicellular vascular structure that separates the central nervous system from peripheral blood circulation, and aging may decrease the stability of the BBB and increase its permeability, potentially increasing the risk of various neurological diseases due to leakage and accumulation of blood components in the brain parenchyma and blood vessel walls [33,34]. However, regular exercise can decrease the permeability of the BBB through increased antioxidant capacity, decreased OS, and anti-inflammatory effects [35]. Moreover, Stamatovic et al., reported that SIRT-1 is an upstream regulator of several ongoing processes in senescent cells and the BBB, and that increased expression or activity of SIRT-1 could be effective in preventing aging-related BBB pathology [36].

Regular exercise can also reduce the production of ROS through regulation of redox status and increase BDNF and VEGF levels, which can promote cell proliferation and growth factors or neurotrophic factors [25,27,37]. These effects can reduce the aging-related structural/functional changes of the brain, thereby preventing cognitive decline such as learning and memory. Meanwhile, a recent study suggested that SIRT-1 can improve brain cognitive function by regulating the expression of BDNF [38].

In this study, after 12 weeks of exercise training, the levels of serum S100β, a blood biomarker reflecting increased BBB damage and permeability, decreased in the Young-Old group, while the levels of BDNF and VEGF increased. These results are hypothesized to be related to the increase in SOD and SIRTs shown in this study; that is, exercise training is effective in increasing neurotrophic factors and decreasing BBB permeability in the elderly, which is thought to be influenced by increased antioxidant capacity and SIRTs. In addition, in this study, the positive effect of exercise training was greater in the relatively younger participants. Bouzid et al., reported that aging changes antioxidant gene transcription factors such as nuclear factor kappa-B and activator protein-1, and that these changes could affect the adaptation effect of exercise by inducing post-transcriptional modifications that could impair the adaptation of antioxidant enzymes on the basis of regular physical activity [21]. Therefore, the results of our study suggest that the activity and response or adaptation of antioxidant enzymes may appear differently depending on the stage of aging, and that the effect of regular exercise may be greater in participants of relatively younger age. We believe this study has the strength to prove that regular exercise can have a positive effect on the functional and structural aspects of the brain by inducing an increase in neurotrophic factors and a decrease in BBB permeability in the elderly, and that the effect may be greater in the younger elderly. However, the study was limited to elderly women, and caloric intake and energy consumption of the participants could not be measured during the study period. Furthermore, Chen et al., reported that exercise training can have a positive effect on cognition and executive function in elderly, but the effect may differ depending on the age of the elderly [39]. In addition, considering that the upregulation of neurotrophic factors through exercise training can benefit the cognitive function of the elderly [40,41], it is suggested that further studies need to verify changes in cognitive function.

## 5. Conclusions

In conclusion, exercise training can be effective in increasing the levels of neurotrophic factors and reducing BBB permeability in the elderly women, and these results are thought to be related to the enhancement of antioxidant capacity and increase in SIRTs. However, the effect of exercise on age-related structural/functional changes in the brain may be greater in Young-Old participants.

## Figures and Tables

**Table 1:** Baseline biochemical and anthropometric characteristics of participants.

Variables	Young-Old (*n* = 12)	Old-Old (*n* = 12)	*p* Value ^#^
Age (years)	69.17 ± 2.55	78.50 ± 1.68	<0.001
Height (cm)	153.96 ± 5.67	152.83 ± 4.06	0.582
Weight (kg)	58.40 ± 5.11	57.02 ± 6.39	0.564
BMI (kg/m^2^)	24.63 ± 1.94	24.38 ± 2.00	0.752
ROS (UM)	195.95 ± 51.95	195.76 ± 41.03	0.992
SOD (U/mL)	6.48 ± 1.65	6.54 ± 2.71	0.940
BDNF (ng/mL)	1.54 ± 0.20	1.47 ± 0.25	0.417
VEGF (pg/mL)	54.00 ± 7.02	52.33 ± 10.95	0.661
S100β (pg/mL)	241.95 ± 55.33	243.01 ± 61.69	0.965
MMP-9 (pg/mL)	4161.69 ± 390.42	4434.72 ± 290.98	0.065
SIRT-1 (ng/mL)	5.54 ± 1.05	5.53 ± 0.97	0.970
SIRT-2 (ng/mL)	1.65 ± 0.07	1.67 ± 0.11	0.471
SIRT-3 (pg/mL)	27.60 ± 6.19	27.09 ± 4.86	0.825

Values presented by mean and standard deviation. BMI, body mass index; ROS, reactive oxygen species; SOD, superoxide dismutase; BDNF, brain-derived neurotrophic factor; VEGF, vascular endothelial growth factor; S100β, S100 calcium-binding protein β; MMP-9, matrix metalloproteinases-9; SIRT-1, sirtuin-1; SIRT-2, sirtuin-2; SIRT-3, sirtuin-3; # *p* value as determined using the independent *t*-test for each of the two groups at baseline.

## Data Availability

Data generated and analyzed during this study are included in this article. Additional data are available from the corresponding author on request.
